# 2D Visualization of the Psoriasis Transcriptome Fails to Support the Existence of Dual-Secreting IL-17A/IL-22 Th17 T Cells

**DOI:** 10.3389/fimmu.2019.00589

**Published:** 2019-04-04

**Authors:** Stephanie T. Le, Alexander A. Merleev, Guillaume Luxardi, Michiko Shimoda, Iannis E. Adamopoulos, Lam C. Tsoi, Jenny Z. Wang, Claire Alexanian, Siba P. Raychaudhuri, Samuel T. Hwang, Johann Gudjonsson, Alina I. Marusina, Emanual Maverakis

**Affiliations:** ^1^Department of Dermatology, University of California, Davis, Sacramento, CA, United States; ^2^Division of Rheumatology, Allergy and Clinical Immunology, Department of Internal Medicine, University of California, Davis, Sacramento, CA, United States; ^3^Department of Dermatology, University of Michigan, Ann Arbor, MI, United States; ^4^Department of Biostatistics, Center for Statistical Genetics, University of Michigan, Ann Arbor, MI, United States; ^5^Albert Einstein College of Medicine, Bronx, NY, United States; ^6^Georgetown University School of Medicine, Washington, DC, United States; ^7^Department of Veterans Affairs, VA Sacramento Medical Center, Northern California Health Care System, Mather, CA, United States

**Keywords:** IL17, IL22, machine learning, neutrophil, psoriasis, RNA-seq, T cell, transcriptome

## Abstract

The present paradigm of psoriasis pathogenesis revolves around the IL-23/IL-17A axis. Dual-secreting Th17 T cells presumably are the predominant sources of the psoriasis phenotype-driving cytokines, IL-17A and IL-22. We thus conducted a meta-analysis of independently acquired RNA-seq psoriasis datasets to explore the relationship between the expression of *IL17A* and *IL22*. This analysis failed to support the existence of dual secreting IL-17A/IL-22 Th17 cells as a major source of these cytokines. However, variable relationships amongst the expression of psoriasis susceptibility genes and of *IL17A, IL22*, and *IL23A* were identified. Additionally, to shed light on gene expression relationships in psoriasis, we applied a machine learning nonlinear dimensionality reduction strategy (t-SNE) to display the entire psoriasis transcriptome as a 2-dimensonal image. This analysis revealed a variety of gene clusters, relevant to psoriasis pathophysiology but failed to support a relationship between *IL17A* and *IL22*. These results support existing theories on alternative sources of IL-17A and IL-22 in psoriasis such as a Th22 cells and non-T cell populations.

## Introduction

Psoriasis is a chronic inflammatory skin condition with nail and systemic manifestations that affects ~3% of the general United States population. It is commonly associated with psoriatic arthritis and is likely linked to other comorbidities, such as cardiovascular disease and metabolic syndrome (1–4).

Of the many clinical variants, plaque psoriasis (psoriasis vulgaris) is the most common, accounting for ~80–90% of cases (1, 5). It is also the most well-characterized histologically and genetically. Plaque psoriasis was initially proposed to be driven by hyperproliferative keratinocytes. However, in 1890, neutrophil involvement was suggested after histologic evaluation revealed early neutrophil accumulation within the dermis and epidermis (i.e., microabscesses of Munro and pustules of Kogoj, respectively) (6).

Despite the clear existence of neutrophils in lesional skin, the role of the adaptive immune system in psoriasis pathophysiology became the main focus of the field after the T cell-targeting agent, cyclosporine, was shown to be an effective treatment (7–9). Thus, psoriasis researchers became very quickly focused on characterizing CD4^+^ and CD8^+^ T cell responses in normal and diseased human skin (10–12). Subsequently, experiments performed in animal models were also developed that supported the T cell-centric view of psoriasis. For example, it was demonstrated that a psoriasis-like phenotype could be induced following adoptive transfer of dysregulated CD4^+^ T cells (13). With this knowledge came the development of the next generation of T cell-targeting therapeutics (alefacept, efalizumab) (14–17), which further corroborated the essential role of T cells in psoriasis pathophysiology.

At the time T cells became the focus of psoriasis, adaptive immune responses were typically divided into two types, T helper type 1 (Th1) and T helper type 2 (Th2) responses. In psoriasis, the absence of Th2-defining cytokines [interleukin (IL)-4, IL-5, and IL-10] (18) and the increased presence of Th1 cytokines (interferon gamma (IFN-γ), tumor necrosis factor (TNF) and IL-12) prompted researchers to classify psoriasis as a Th1-mediated disease (18). Soon thereafter, however, it became increasingly apparent that IL-17-secreting T cells (Th17 cells) played a major role in disease pathogenesis, not only in psoriasis, but also across a wide spectrum of animal models of autoimmunity (19–22).

Psoriasis is now thought to be a predominantly Th17-driven disease (23, 24) that is maintained by the key Th-17-supporting cytokine, IL-23 (25, 26). The dominant role of the IL-23/IL-17A axis in psoriasis is also evident by the overwhelming clinical success of newly developed IL-23/IL-17A axis-targeting biologics, which could induce near complete resolution of psoriasis, even in the most severely affected individuals (27–29). IL-22 is also a highly investigated cytokine involved in psoriasis pathophysiology. It is thought to be the primary promoter of keratinocyte hyperproliferation (30, 31). The predominant view is that this cytokine is secreted by IL-17A/IL-22 dual-secreting Th17 cells (32).

However, the observed pathogenicity of IL-17A/IL-22 dual-secreting Th17 cells has never been formally demonstrated *in vivo*. In fact, the vast majority of evidence in support of these cells have come from animal studies and *in vitro* analysis of human T cells cultured under extreme polarizing conditions (32–35). Even when studied directly *ex vivo*, the dual secretion is usually seen only after non-physiologic T cell stimulation (36, 37). Since naturally processed autoimmune epitopes are difficult to identify (38), it is challenging to study cytokine secretion using more physiologic stimuli.

Thus, we sought evidence for the existence of dual secreting IL-17A/IL-22 Th17 cells within the psoriasis transcriptome. Weighted gene co-expression networks analysis (WGCNA) (39) have previously been used to analyze gene-gene correlations within RNA-Seq datasets. While this strategy has certain advantages, it is not ideally suited to explore gene relationships across multiple RNA-Seq datasets. Herein, we conduct meta-analyses of RNA-seq datasets to directly evaluate the current hypothesis that dual-secreting IL-17A/IL-22 Th17 cells are the dominant effector population in psoriasis. We also used this strategy to correlate the expression of *IL17A, IL22*, and *IL23A* with genes linked to psoriasis susceptibility identified through genome-wide association studies (GWAS). Finally, to explore the gene expression profile of *IL17A, IL22*, and *IL23A* in relation to other genes expressed in psoriatic plaques, we utilized a machine learning nonlinear dimensionality reduction strategy to visualize the entire psoriasis transcriptome as a 2-dimensional (2D) image. This allowed us to clearly visualize the relationship between *IL17A, IL22*, and *IL23A* and all other genes that are expressed in psoriatic skin.

## Materials and Methods

### Human RNA-Seq

RNA-Seq FASTQ files of human normal and psoriasis lesional skin were downloaded from the NCBI Sequence Read Archive (http://www.ncbi.nlm.nih.gov/Traces/sra). Four total datasets were used: Three datasets (Accession numbers: SRP165679, SRP026042, SRP057087) and one dataset comprised of two combined experimental datasets published by the same research group (Accession numbers: SRP035988, SRP050971) (40–42).

### Correlations

Correlation analyses of gene expressions were performed on read counts of each identified gene normalized with DESeq2 package (43). Values were subsequently log transformed and winsorized when necessary. Spearman's correlation coefficients were calculated (r_s_) using the cor.test function in R (44). *P* values of the correlations were estimated by algorithm AS 89.

### 2D Visualization of the Psoriasis Transcriptome

We computed the gene pairwise distance using a formula, 1-r^2^, where r represents Pearson's correlation. A visual representation of the gene co-expression network was created using a dimensionality reduction technique, t-Distributed Stochastic Neighbor Embedding (t-SNE), calculated with Rtsne package (45).

### Gene Selection

A Pubmed search was performed to identify genes linked to psoriasis through GWAS.

Genes selected for mapping included: *BTK, CD3E, CD4, CD8a, CD8b, CD19, CTSG, CXCL1, CXCL2, CXCL5, ELANE, ICAM1, IGH, IGK, IGL, IL1B, IL8, IL17A, IL22, IL23A, IL36A, IL36B, IL36G, IFNG, ITK, MPO, MS4A1* (CD20), *TNF, TRA* (TCRα), *TRB* (TCRβ)*, TRD* (TCRδ) *TRG* (TCRγ).

Genes selected for meta-analysis included: *B3GNT2, CARD14, CARM1, CDKAL1, CTSG, CXCL1, CXCL5, CXCR2, DDX58, DEFB4A, ELANE, FBXL19, GJB2, HLAC, IFIH1, IL12B, IL17A, IL22, IL23A, IL36RN, IL4R, KLF4, KRT1, KRT5, KRT6A, KRT6B, KRT6C, KRT10, KRT14, KRT16, KRT17, KRT37, LCE3A, LCE3B, LCE3D, MPO, NFKBIA, NOS2, NOS3, PTPN22, RELB, RUNX3, SOCS1, STAT3, STAT5A, TNFAIP3, TNFRSF9, TNIP1, TRAF3IP2, TYK2, UBE2L3, VDR, VEGFA, VEGFB*.

### Meta-Analysis

Meta-analysis was completed using the R package “metafor” (46). A weighted random-effects model was used to estimate a summary effect size. To estimate between-study variance, a restricted maximum-likelihood estimator was applied. A weighted estimation with inverse-variance weights was used to fit the model.

## Results

### The Expression of *IL17A* and *IL22* Do Not Strongly Correlate With One Another in Psoriatic Plaques

We hypothesized that if a significant amount of IL-17A and IL-22 is produced by IL-17A/IL-22 dual secreting Th17 cells in psoriasis, then the gene expression of these two cytokines should correlate with one another. In theory, their expression would be directly linked to the number of dual-secreting Th17 cells in a psoriasis plaque. Their gene expression should also correlate with *IL23A*, which activates and maintains Th17 cells.

**Figure 1:**
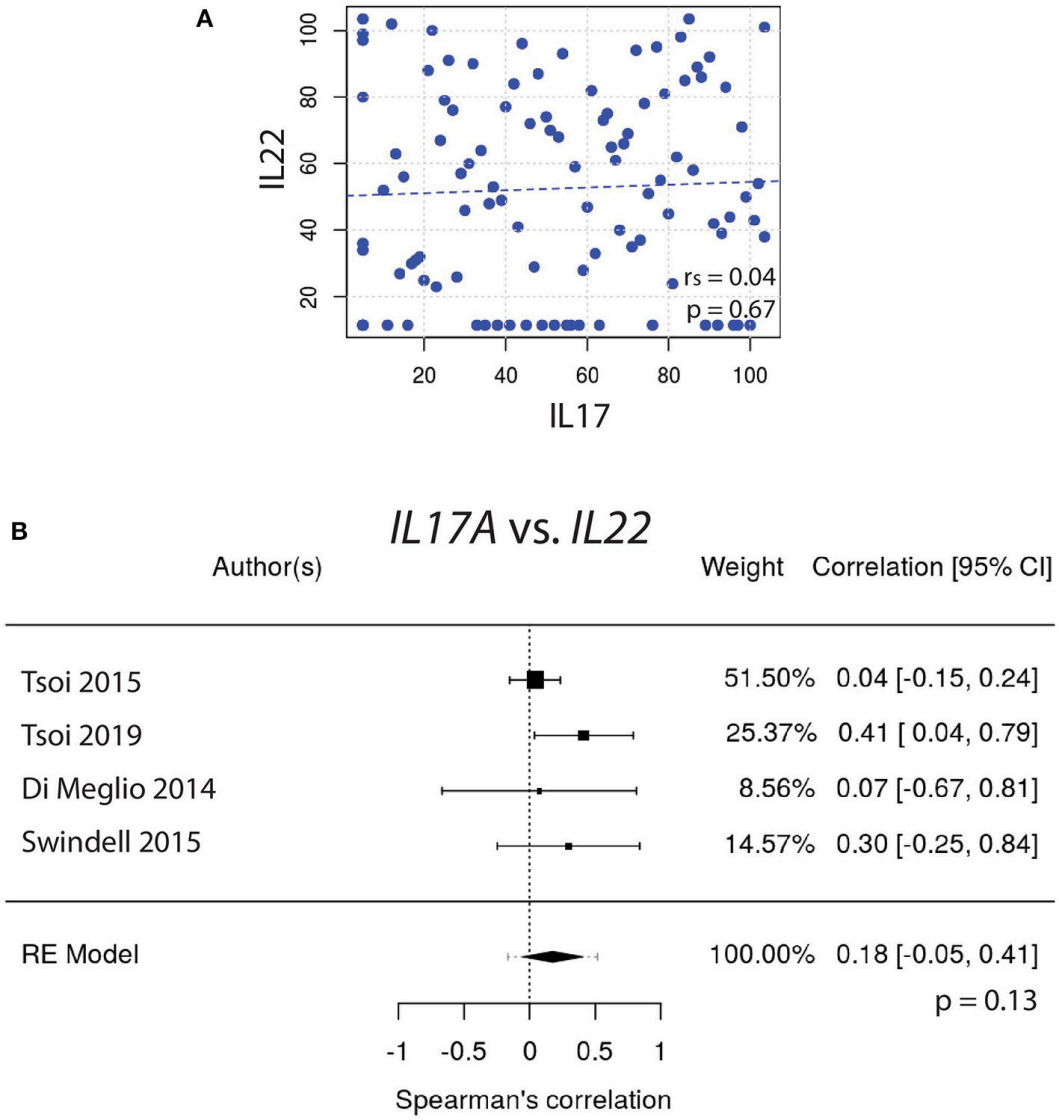
**(A)** The expression of *IL17A* does not strongly correlate with the expression of *IL22* (r_s_ = 0.04). **(B)** Meta-analysis of four datasets further supports that *IL17A* and *IL22* expression do not strongly correlate [r_s_ = 0.18, with a confidence interval that crosses 0 (−0.05, 0.41)].

To test this hypothesis, gene expression of *IL17A* vs. *IL22* was graphed and the Spearman's correlation coefficient (r_s_) was calculated (Figure 1A). These correlative studies demonstrated that the expression of *IL22* does not strongly correlate with the expression of *IL17A* (r_s_ = 0.04, *p* = 0.67). To obtain a weighted average across all four independently acquired psoriasis datasets, a meta-analysis was performed and the resulting Forest plot (Figure 1B) demonstrated again that *IL17A* and *IL22* do not strongly correlate with one another [r_s_ = 0.18, with a confidence interval that crosses 0 (−0.05, 0.41)] (Supplemental Figure 1).

**Figure 2:**
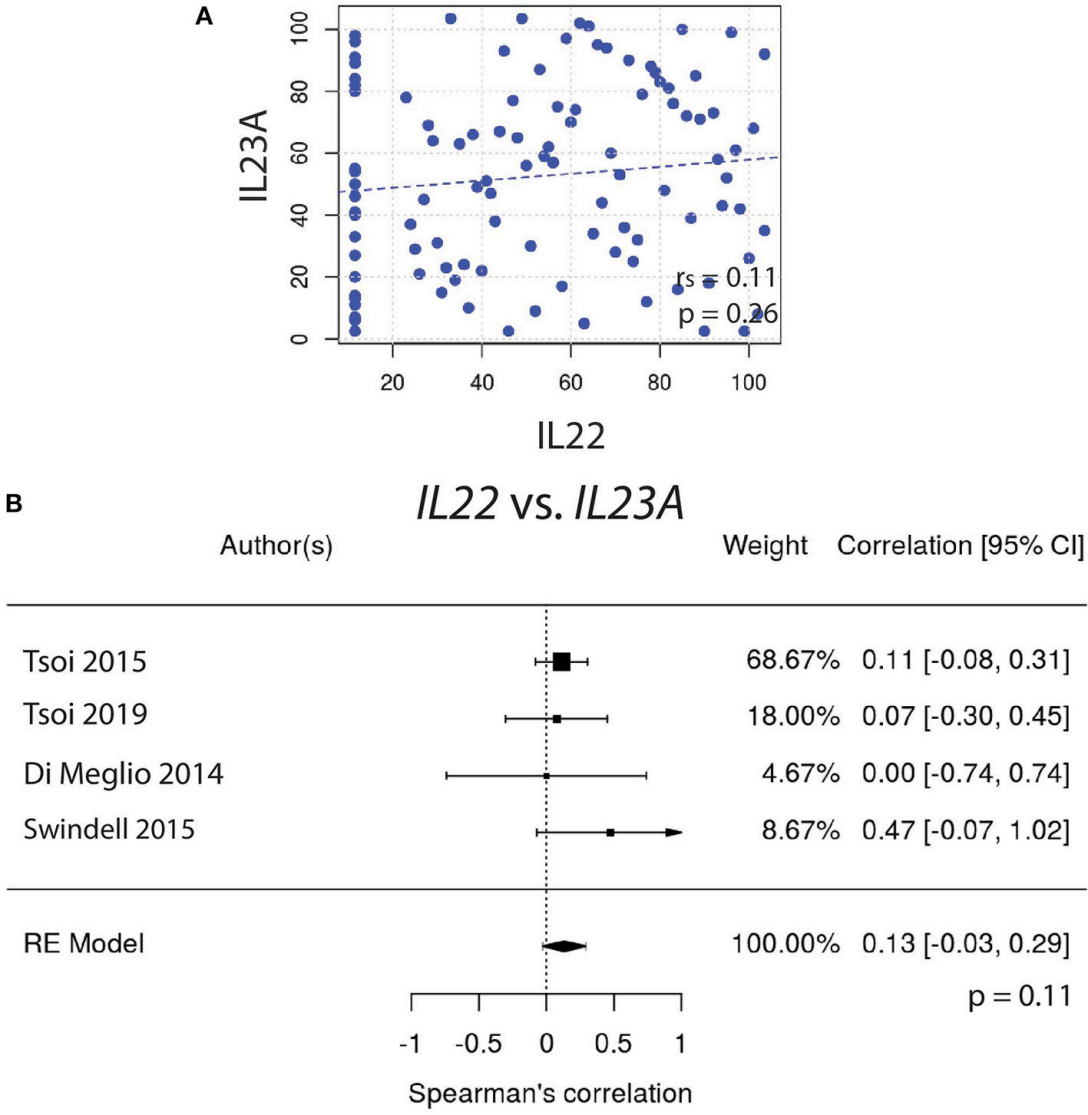
**(A)** *IL22* gene expression does not correlate with *IL23A* (r_s_ = 0.11). **(B)** Meta-analysis confirms that *IL22* and *IL23A* do not strongly correlate [r_s_ = 0.13, with a confidence interval that crosses 0 (−0.03, 0.29)].

**Figure 3:**
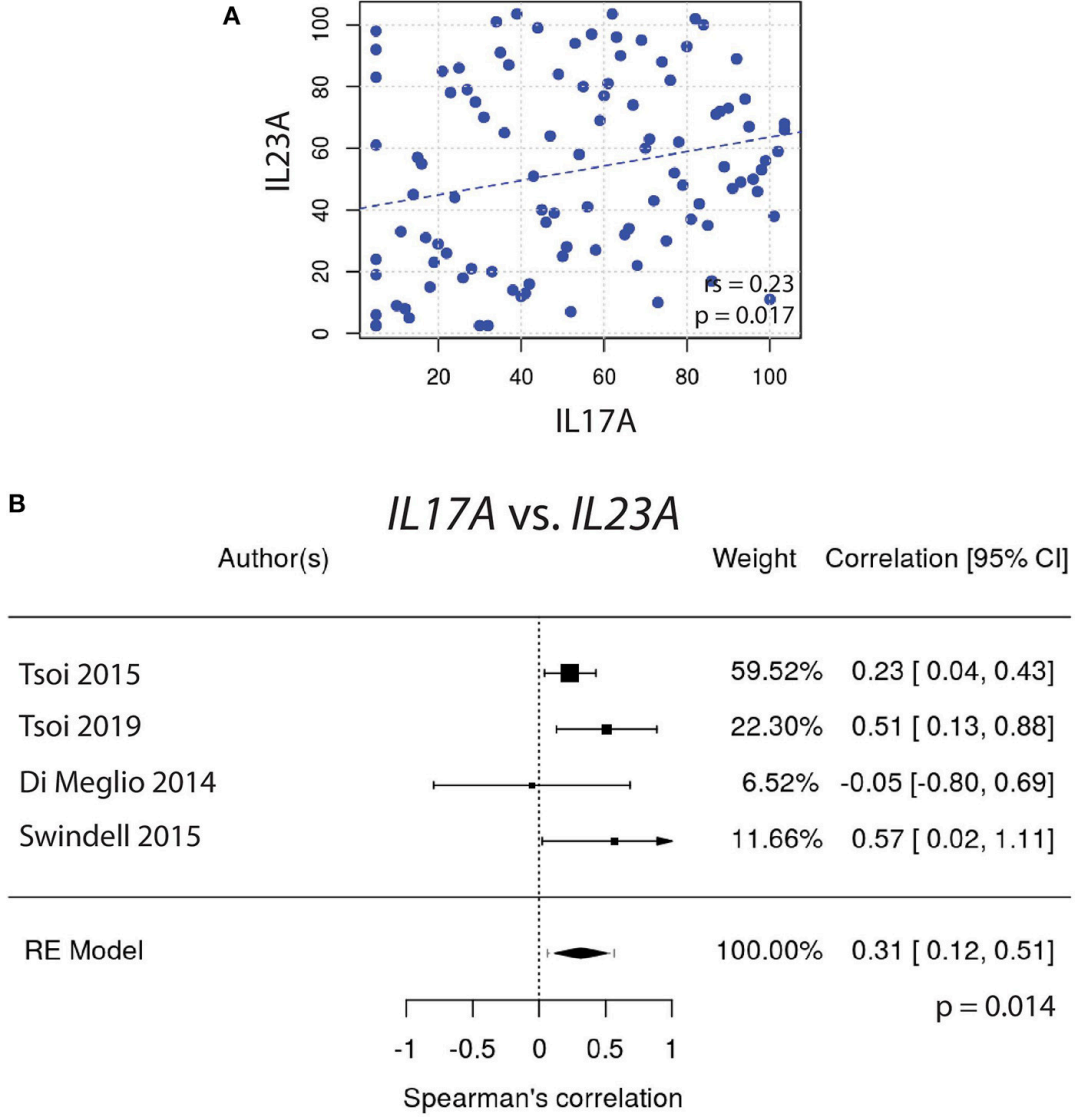
**(A)** A significant Spearman correlation (r_s_ = 0.23, *p* = 0.017) is seen between *IL17A* and *IL23A* gene expression. **(B)** Meta-analysis supports a strong correlation between *IL17A* and *IL23A* [r_s_ = 0.31, (0.12, 0.51); *p* = 0.0014].

Similarly, *IL22* gene expression did not correlate well with *IL23A* (Figures 2A,B). In contrast, the expression of *IL17A* did correlate very well with *IL23A* (Figure 3A), a result that was consistent amongst a majority of datasets. The weighted average of this correlation across all psoriasis datasets was highly significant, [r_s_ = 0.31 (0.12, 0.51); *p* = 0.0014], with no evidence (p = 0.33) of any substantial residual heterogeneity (i.e., there was no remaining variability in effect sizes that was unexplained) (Figure 3B). Our data confirms the well-characterized dependency of IL-17A on IL-23A. However, IL-22 was not found to have a similar dependency on IL-23A, casting doubt on the theory that IL-17A and IL-22 are secreted mainly by the same dual-secreting cell.

### 2D Visualization of the Psoriasis Transcriptome Reveals T Cell, B Cell, Inflammatory Cytokines, Neutrophil-Recruiting, and Neutrophil Gene Clusters

To determine how genes expressed in psoriatic plaques are related to one another, correlation coefficients were calculated for all pairwise comparisons. The distances between each gene pair was calculated as described in the methods. The resulting distance matrix was then used to construct a 2D image using t-SNE.

**Figure 4:**
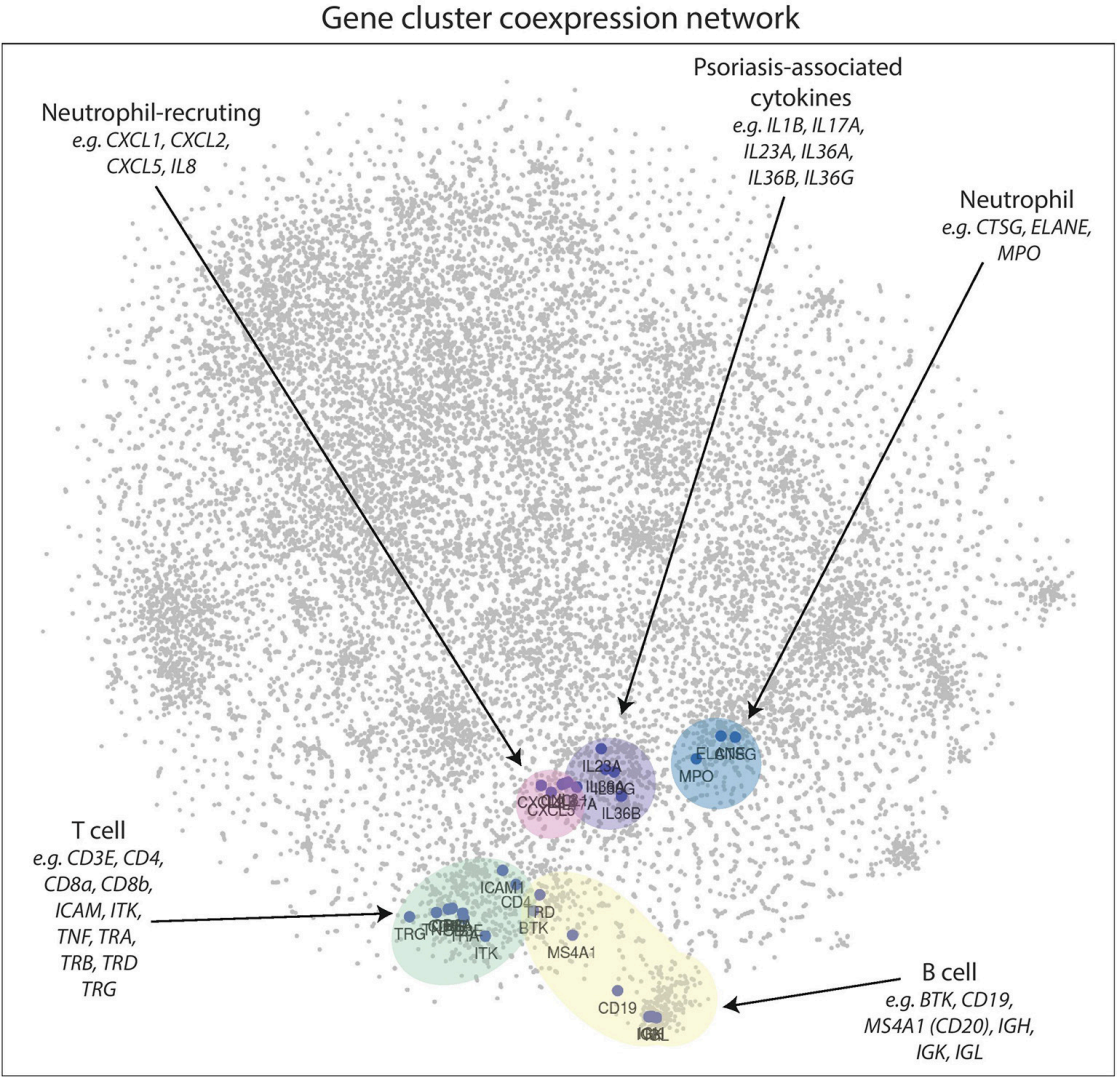
2D gene coexpression network illustrates gene expression relationships of the psoriasis plaque. Points closer together represent genes that have higher correlation coefficients, while genes at further distances generally do not correlate well. Gene clusters appear to correspond to neutrophil-recruiting cytokines, neutrophil effector molecules, T cell, B-cell, and psoriasis-associated genes in lesional psoriatic skin.

In the 2D plot (Figure 4), genes that highly correlate with one another tend to be located in the same region, known as a cluster. Genes that do not cluster near each other do not correlate well. Figure 4 clearly demonstrates that genes associated with B cells [*BTK, CD19, IGH, IGK, IGL, MS4A1* (CD20)], T cells (*CD3E, CD4, CD8a, CD8b, ICAM1, ITK, TRA TRB, TRD, TRG*), neutrophils (*CTSG, ELANE, MPO*), neutrophil-recruiting (*CXCL1, CXCL2, CXCL5, IL8*), and psoriasis-associated inflammatory cytokines (*IL1B, IL17A, IL23A, IL36A, IL36B, IL36G*) cluster well together in distinct groups, which supports this method as a means to visualize the entire psoriasis transcriptome.

### *IL22* Does Not Cluster With Other Inflammatory Cytokines Involved in Psoriasis, Including *IL17A*

**Figure 5:**
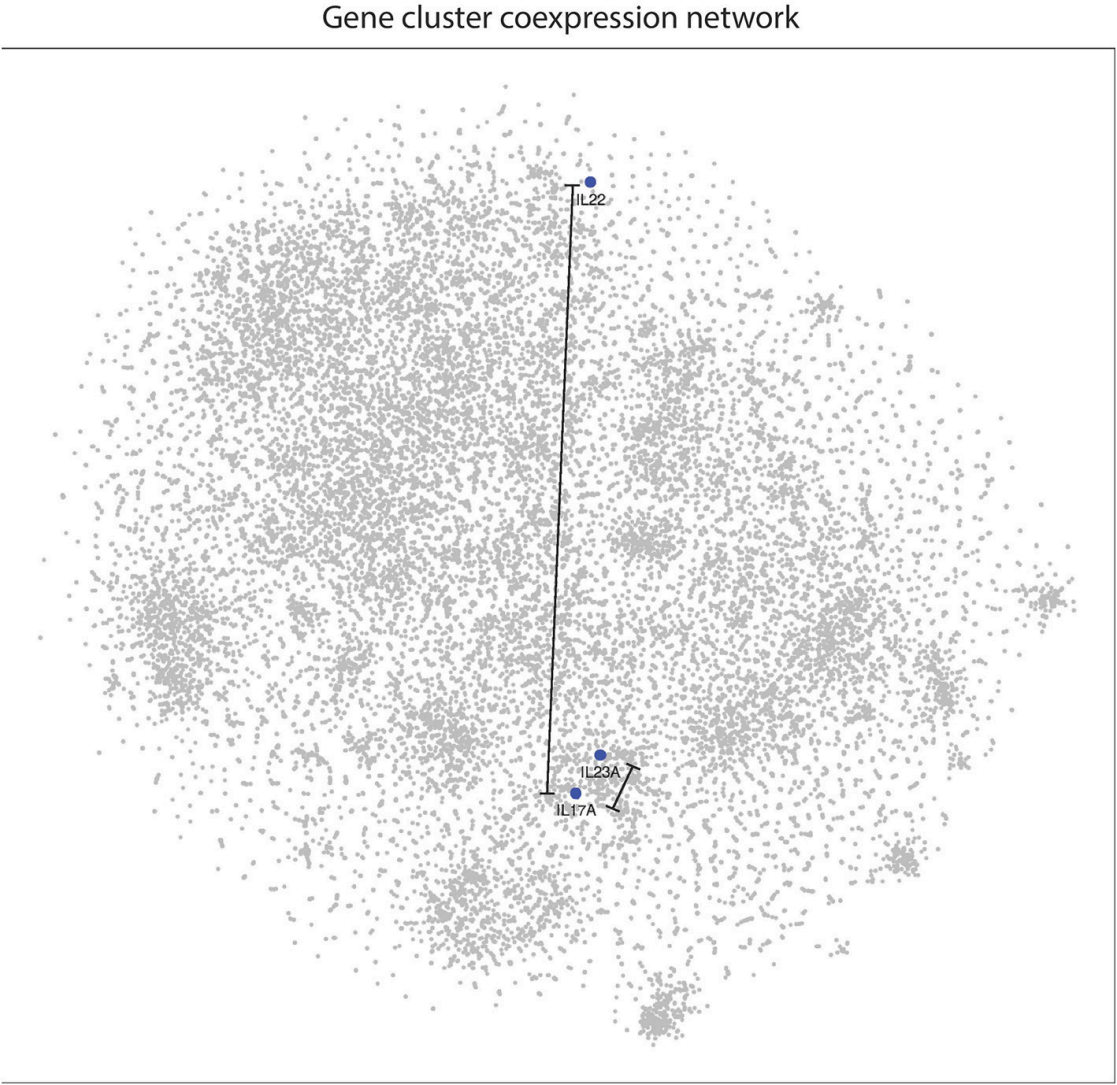
*IL22* is located at a relatively great distance away from *IL17A* and *IL23A* in the psoriasis transcriptome, as illustrated in the 2D gene expression network map of psoriasis. These results suggest that *IL22* is not highly linked to either *IL17A* or *IL23A* in psoriasis pathophysiology.

With respect to other cytokines and chemokines involved in psoriasis pathophysiology, *IL22* is located peripherally at a relatively great distance away on the 2D plot of the psoriasis transcriptome (Figure 5). This supports our results from the meta-analyses and suggests that *IL22* does not correlate well with *IL23A*. Interestingly, *IL22* does not cluster well with any of the most commonly implicated cytokines in psoriasis pathophysiology. In contrast, *IL17A* clusters together with *IL23A* and the other cytokines thought to be involved in psoriasis pathophysiology.

### *IL22* Correlates With Keratins

Several studies have demonstrated that IL-22 stimulates keratinocytes. There is a variety of evidence, including data obtained from *in vitro* studies with skin-like organoid cultures, that support IL-22 as the main cytokine responsible for epidermal hyperplasia, a hallmark of psoriasis (47, 48).

**Figure 6:**
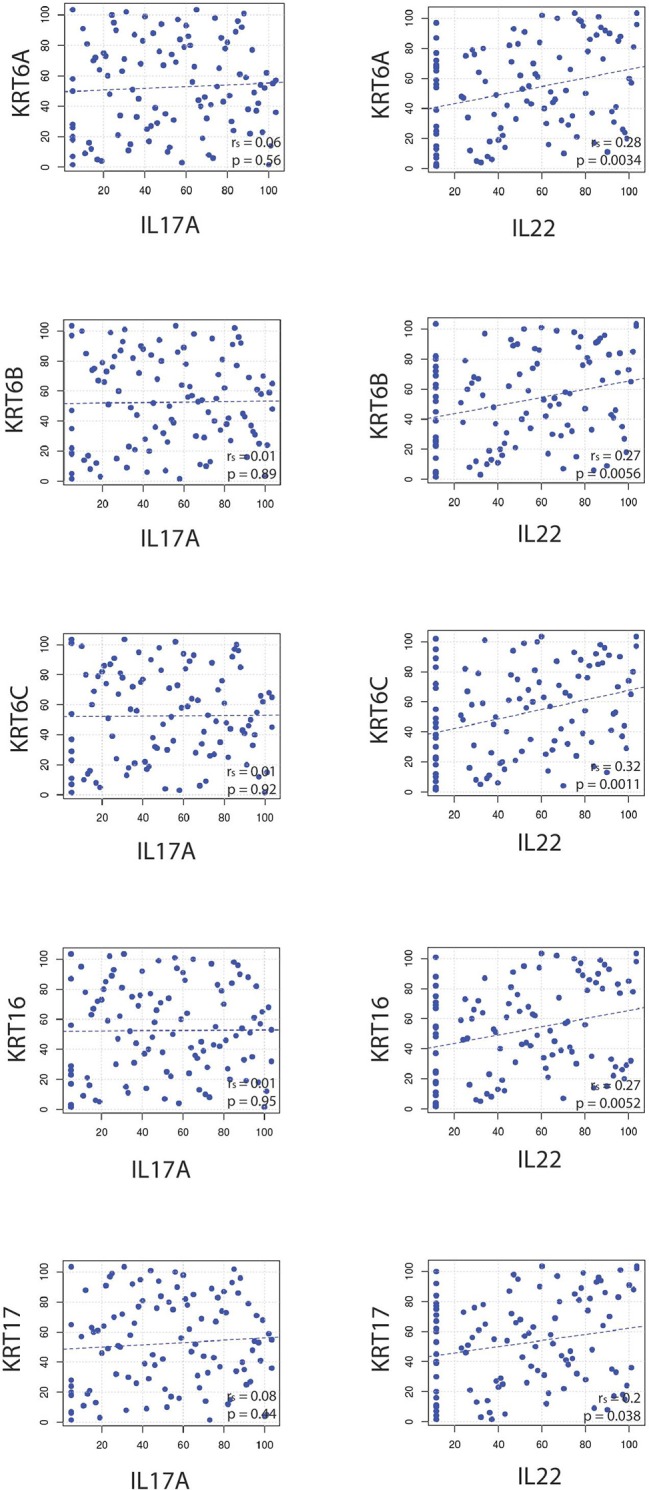
Correlative studies demonstrate that *IL22* strongly correlates with commonly psoriasis-associated keratin genes (*KRT6A, KRT6B, KRT6C, KRT16, KRT17*), a finding that was not true for *IL17A*.

**Figure 7:**
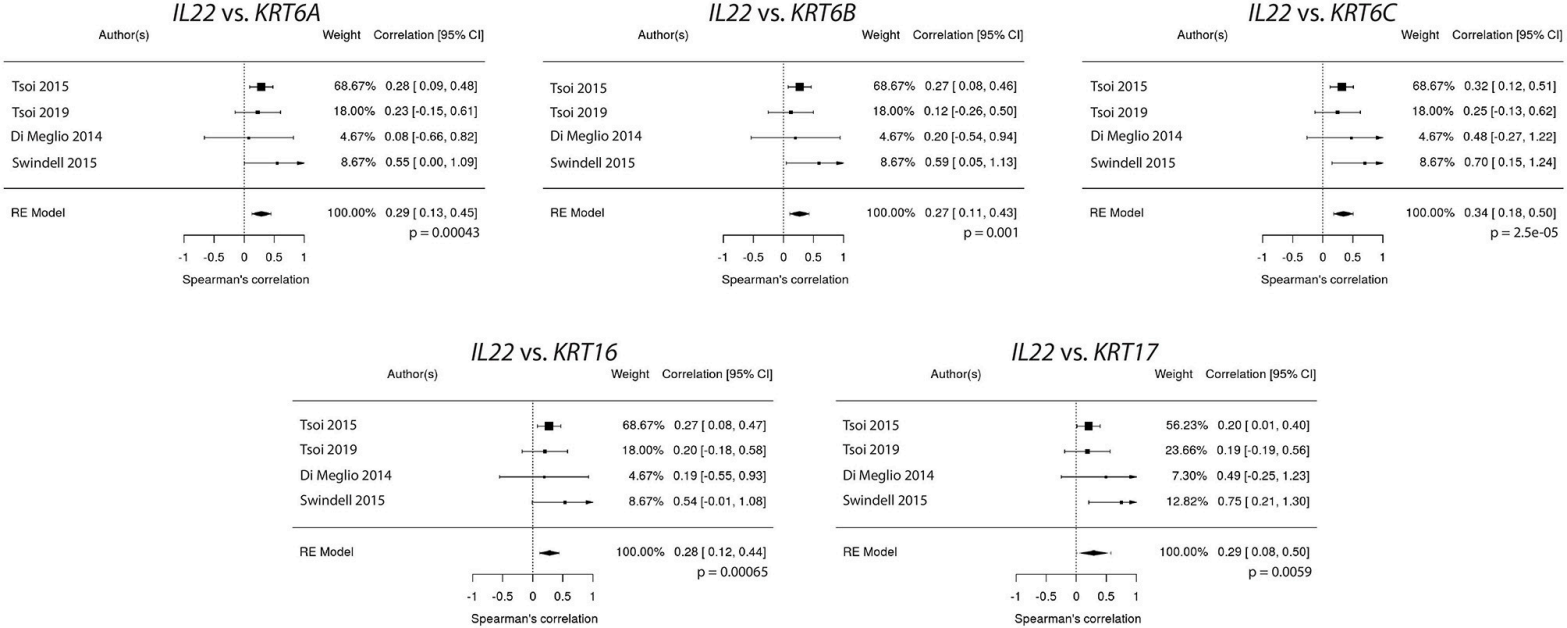
Meta-analysis confirms a close relationship between *IL22* expression and keratin (*KRT6A, KRT6B, KRT6C, KRT16, KRT17*) expression.

Because *IL22* failed to strongly correlate with *IL17A* and *IL23A* (Figures 1A, 2A), the relationships between *IL22* and keratin genes were explored across the four independently acquired RNA-Seq psoriasis datasets. Again, Spearman's correlation coefficients (r_s_) were calculated for each keratin gene's relationship with *IL17A, IL22*, and *IL23A*. These correlative studies demonstrate that the expression of *IL22* did indeed strongly correlate with the expression of the different keratin genes (Figure 6), especially *KRT6C* (keratin 6C) (r_s_ = 0.32, *p* = 0.0011). To obtain a weighted average across all four independent psoriasis datasets, a meta-analysis was performed and the resulting Forest plots (Figure 7) confirm the close relationship between *IL22* expression and keratin gene expression [*KRT6C*: r_s_ = 0.34, with a confidence interval that did not cross 0 (0.18–0.50)]. The weighted average of this correlation across all psoriasis datasets was highly significant (*p* = 0.000025), with no evidence (*p* = 0.56) of any substantial residual heterogeneity (i.e., there was no remaining variability in effect sizes that was unexplained). Additional genes that were found to positively correlate with *IL22* expression are listed in Supplemental Figure 2.

In contrast to *IL22*'s relationship with keratin gene expression, *IL17A* did not correlate well with the keratins (Figure 6), a finding that was confirmed by a meta-analysis across all four RNA-Seq datasets.

### *IL23A* Correlates With Other Genes Besides *IL17A*

**Figure 8:**
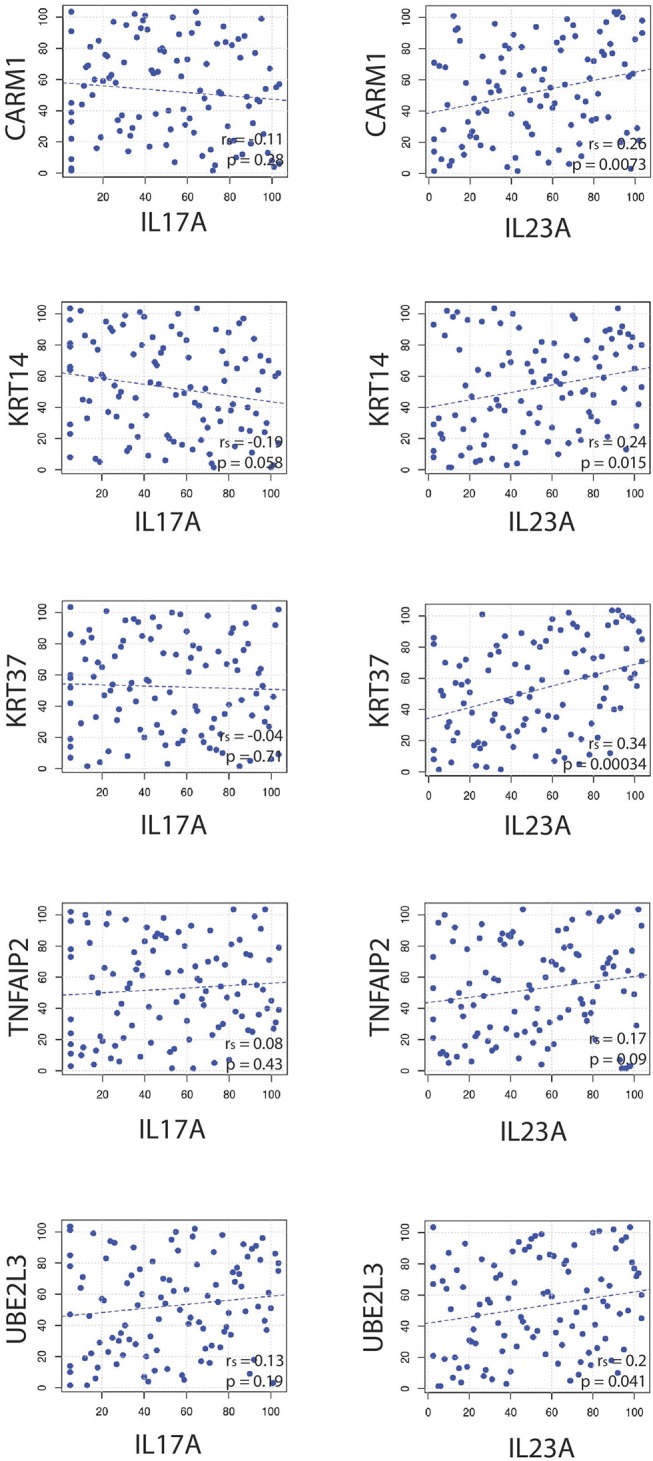
Spearman correlations reveal that *IL23A* correlates with several genes that do not seem to be strongly related to *IL17A*.

IL-23A is known for its ability to support Th17 T cells but it likely has a variety of functions independent of this role. To investigate this, *IL23A*'s ability to independently correlate with other immune-relevant genes was explored. Figure 8 reveals that *IL23A* correlates with several genes unrelated to *IL17A*, a finding confirmed by meta-analyses across all psoriasis RNA-Seq datasets. Included in the analysis were genes identified by GWAS to be linked to psoriasis. Of these genes, *CARM1, KRT14, KRT37, TNFAIP3, UBE2L3* are elevated in psoriasis plaques compared to control healthy skin (Table 1). Thus, *IL23A* appears to be linked to other genes putatively involved in the pathophysiology of psoriasis that are unrelated to *IL17A*.

**Table 1:** Expression of genes linked to psoriasis identified through GWAS and associated *IL23A* Spearman correlation and *p*-value and fold change increase in lesional skin and *p*-value.

**Gene**	***IL23A* Spearman correlation**	***IL23A* Spearman *P*-value**	**Fold change increase in lesional skin**	**Fold change increase *P*-value**
*B3GNT2*	−0.16	0.10537	1.001155781	0.979956721
*CARD14*	0.27	0.00682	2.389097546	1.33E−47
*CARM1*	0.26	0.00968	1.141422929	0.000834388
*CDKAL1*	−0.21	0.03518	0.8683625	3.52E−08
*CTSG*	−0.16	0.10656	0.655654701	4.50E-13
*CXCL1*	0.46	1.30E−06	83.27818074	2.62E−212
*CXCL5*	0.34	0.00056	26.29025755	2.84E−33
*CXCR2*	0.52	6.60E−08	6.492199066	1.40E−239
*DDX58*	0.33	0.00092	2.797571851	1.14E−69
*DEFB4A*	0.35	0.00034	1901.591548	2.62E−271
*ELANE*	−0.0041	0.96819	0.491347624	6.77E−10
*FBXL19*	0.34	0.00062	1.85489088	5.24E−28
*GJB2*	0.48	4.30E−07	20.36794869	0
*HLAC*	0.18	0.07342	1.194566942	0.001439986
*IFIH1*	0.42	1.40E−05	3.162866511	7.72E−104
*IL12B*	0.29	0.00397	29.88723345	3.40E−57
*IL17A*	0.24	0.01769	439.3348171	1.25E−65
*IL22*	0.1	0.31571	63.88350813	3.46E−30
*IL23A*	1	< 2E−16	3.760844628	8.35E−45
*IL36RN*	0.48	4.90E−07	7.305421524	1.16E−230
*IL4R*	0.51	6.60E−08	3.093054089	8.95E−181
*KLF4*	0.012	0.90338	0.667068955	3.81E−15
*KRT1*	−0.012	0.90464	1.206311292	0.001401592
*KRT10*	−0.062	0.54239	1.110563157	0.047606741
*KRT14*	0.22	0.02787	1.536561931	1.27E−11
*KRT16*	0.27	0.00767	45.90558693	0
*KRT17*	0.39	6.00E−05	4.076992497	2.38E−54
*KRT37*	0.3	0.00255	5.231342887	2.43E−46
*KRT5*	0.094	0.35243	1.303825713	1.61E−07
*KRT6A*	0.39	8.30E−05	24.37406248	0
*KRT6B*	0.29	0.00374	8.511861588	5.34E−107
*KRT6C*	0.25	0.01245	117.818558	3.29E−294
*LCE3A*	0.39	6.50E−05	184.4450577	6.18E−144
*LCE3B*	0.2	0.04403	67.28771597	2.14E−38
*LCE3D*	0.31	0.00208	36.65620978	0
*MPO*	−0.24	0.01605	1.474609904	0.0051495
*NFKBIA*	0.31	0.00184	1.194928571	6.14E−07
*NOS2*	0.64	1.20E−12	45.89643372	5.06E−181
*NOS3*	−0.024	0.81219	1.120615697	0.065873995
*PTPN22*	0.09	0.38043	2.512943835	2.58E−45
*RELB*	0.3	0.0023	1.63227558	3.66E−17
*RUNX3*	0.073	0.47497	0.879564111	0.003653355
*SOCS1*	0.41	3.10E−05	1.851306719	1.98E−17
*STAT3*	0.51	6.50E−08	2.199106208	7.64E−127
*STAT5A*	0.012	0.90974	0.825113995	1.27E−08
*TNFAIP3*	0.825113995	1.27E−08	1.053151539	0.237418045
*TNFRSF9*	0.24	0.01749	6.76780515	2.64E−126
*TNIP1*	0.4	4.20E−05	1.591537669	1.52E−33
*TRAF3IP2*	0.28	0.00626	1.27943256	1.21E−23
*TYK2*	0.084	0.41131	1.10633692	0.027521096
*UBE2L3*	0.22	0.03143	1.247192396	1.13E−20
*VDR*	0.36	0.00025	1.010394091	0.770645743
*VEGFA*	0.092	0.36524	1.300943308	1.11E−08
*VEGFB*	−0.12	0.22006	0.640896258	1.01E−36

### *IL17A, IL22*, and *IL23A* Expression Correlates With Psoriasis Susceptibility Genes

**Figure 9:**
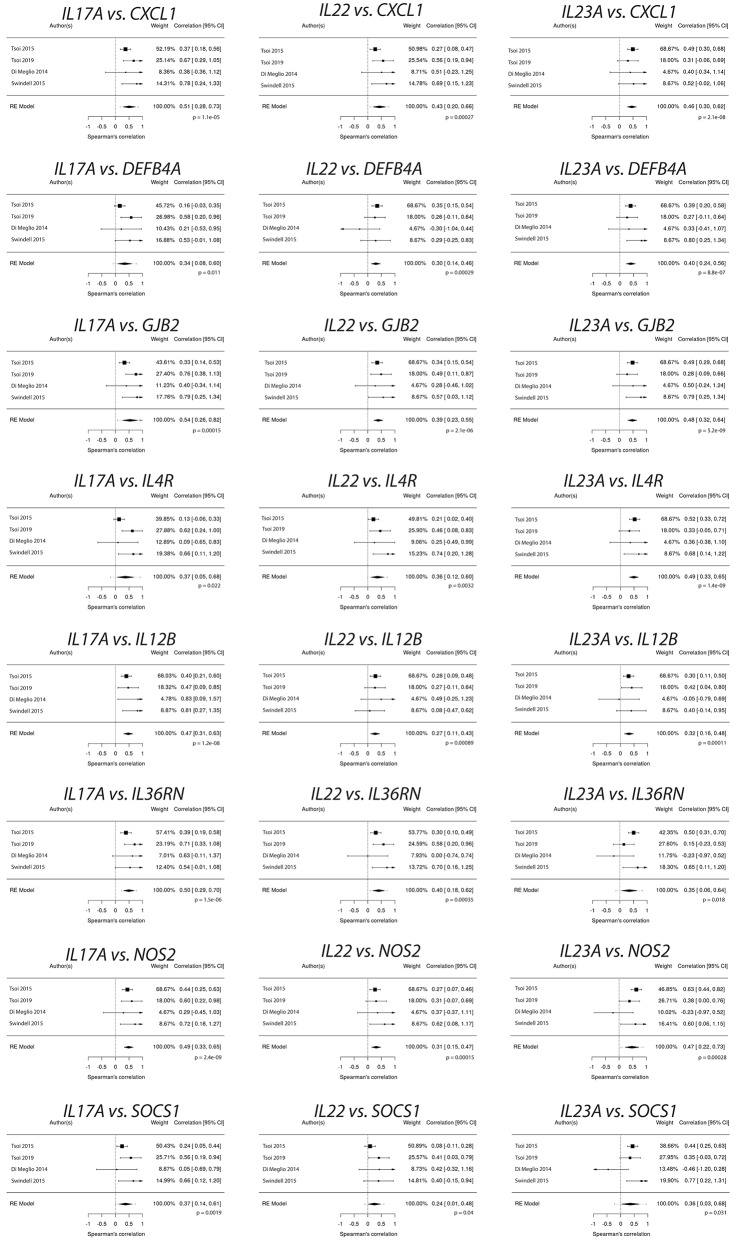
Psoriasis susceptibility genes that positively correlate across *IL17A, IL22*, and *IL23A*, supporting a link between these cytokines and genes that have been linked to psoriasis through GWAS.

A variety of genes have been linked to psoriasis susceptibility through GWAS (49–55). Table 2 demonstrates that many of these genes are differentially regulated in the setting of psoriasis. We thus sought to determine how the expression of genes located at psoriasis susceptibility loci correlated with the expression of *IL17A, IL22*, and *IL23A*, genes known to be linked to the pathophysiology of psoriasis. For this analysis, the expression of *IL17A, IL22*, and *IL23A* was plotted against the expression of each of the genes identified through GWAS studies. Spearman's correlation coefficients (r_s_) were then calculated, which demonstrated a variety of significant correlations (Table 2) between GWAS-identified genes and *IL17A, IL22*, and *IL23A*. Correlation values between atopic dermatitis GWAS-identified genes and *IL17A, IL22* and *IL23A* expression in psoriasis samples were also obtained for comparison (Table 3). To obtain a weighted average across all four independent psoriasis data sets, meta-analysis was performed. The resulting Forest plots are depicted in Figure 9, which confirm the close relationship between *IL17A, IL22*, and *IL23A* and the different genes linked to psoriasis susceptibility. These results support a direct or indirect link between *IL17A, IL22*, and *IL23A* and these genes. Of note, the genes that significantly correlated with *IL17A, IL22*, and *IL23A* varied for each cytokine. These results will hopefully help investigators better understand the pathophysiology of psoriasis.

**Table 2:** Correlation (*R*-values) of *IL17A, IL22*, and *IL23A* with genes linked to psoriasis susceptibility through genome-wide association studies.

	***R*****-value**		
**Gene**	***IL17A***	***IL22***	***IL23A***
*CARD14*	**0.18**	0.16	**0.3**
*CARM1*	−0.08	0.1	**0.26**
*CDKAL1*	−0.2	−0.15	–**0.21**
*DDX58*	**0.35**	0.06	**0.28**
*DEFB4A*	**0.32**	**0.3**	**0.4**
*GJB2*	**0.54**	**0.39**	**0.48**
*HLAC*	−0.07	−0.05	0.02
*IFIH1*	**0.37**	−0.02	**0.34**
*IL12B*	**0.47**	**0.28**	**0.32**
*IL17A*	**1**	0.18	**0.31**
*IL22*	0.18	**1**	0.13
*IL23A*	**0.31**	0.13	**1**
*IL36RN*	**0.5**	**0.4**	**0.35**
*IL4R*	**0.37**	**0.36**	**0.49**
*KLF4*	–**0.36**	−0.03	0
*LCE3A*	**0.21**	**0.26**	0.26
*LCE3B*	−0.03	**0.2**	**0.17**
*LCE3D*	0.08	**0.21**	**0.32**
*NOS2*	**0.49**	**0.31**	**0.47**
*NOS3*	0.04	0.08	0.1
*PTPN22*	**0.49**	0.09	**0.22**
*RELB*	0.03	**0.2**	**0.35**
*RUNX3*	–**0.18**	−0.04	0.13
*SOCS1*	**0.37**	**0.24**	**0.36**
*STAT3*	**0.32**	**0.45**	0.35
*TNFAIP3*	−0.04	0.03	**0.21**
*TNFRSF9*	**0.35**	0.02	**0.3**
*TNIP1*	0.23	**0.18**	0.37
*TRAF3IP2*	0.07	**0.28**	**0.24**
*TYK2*	0	0.06	0.14
*UBE2L3*	0.18	0.06	**0.17**
*VDR*	0.07	**0.17**	**0.4**
*VEGFA*	0.01	**0.23**	**0.2**
*VEGFB*	**−0.25**	**−0.18**	−0.21

*Bolded cells p < 0.05*.

**Table 3:** Correlation (*R*-values) of *IL17A, IL22 and IL23A* in psoriasis with genes linked to atopic dermatitis susceptibility through genome-wide association studies (56–61).

	***R-*****value**		
**Gene**	***IL17A***	***IL22***	***IL23A***
*ADAMTS10*	−**0.16**	−0.05	−0.10
*C11orf30*	**0.16**	−0.07	−0.03
*LRRC32*	−**0.18**	−0.04	−0.11
*CARD11*	0.10	0.05	0.15
*CCDC80*	−**0.24**	−0.05	–**0.22**
*CLEC16A*	0	0.12	**0.38**
*CYP24A1*	**0.32**	0.14	**0.32**
*FLG*	−**0.28**	–**0.16**	–**0.36**
*GLB1*	−0.14	−0.02	−0.02
*GPSM3*	−0.04	0	0.06
*IL18R1*	**0.23**	−0.05	0.02
*IL18RAP*	**0.23**	0.05	**0.27**
*IL2*	0.09	0.01	0.04
*IL6R*	−0.03	−0.10	0.11
*KIF3A*	0.14	−0.02	−0.01
*IL13*	0	**0.26**	0.06
*NLRP10*	−0.17	−0.11	–**0.19**
*OR10A3*	0.08	−0.08	0.08
*OVOL1*	**0.32**	**0.28**	**0.32**
*PFDN4*	0.01	−0.12	−0.06
*PRR5L*	**0.28**	−0.05	−0.06
*RAD50*	**0.20**	0.13	−0.01
*TMEM232*	0	0.11	−0.06
*SLC25A46*	0.06	0.03	−0.07
*TNFRSF6B*	−0.06	0.14	**0.28**
*ZGPAT*	0.03	0.12	**0.18**
*ZNF652*	−**0.38**	–**0.19**	–**0.42**

*Bolded cells p < 0.05*.

## Discussion

Investigators have employed numerous genetic strategies to characterize the immune response in the setting of psoriasis. Microarray and RNA-Seq have provided insight into the psoriatic transcriptome, identifying thousands of differentially expressed genes (40). However, differential expression alone does not necessarily mean that the gene is involved in psoriasis pathogenesis. For example, a gene that is normally downregulated in psoriatic T cells may actually appear falsely upregulated in psoriasis simply because there are more T cells in a psoriatic plaque. With the rising popularity of single cell sequencing, investigators are now focused on re-characterizing the psoriasis transcriptome at a greater cellular resolution, not previously obtained with whole tissue transcriptomics. However, single cell sequencing is also not without its drawbacks. Purifying immune cell populations from skin biopsy specimens can alter their transcriptome, especially for cells isolated by positive selection or flow cytometry. Furthermore, immune cells within the skin will undoubtedly have different purification yields. T cells in particular are especially difficult to analyze because once purified, they require additional non-physiologic *in vitro* stimulation with lectins or anti-CD3/anti-CD28 antibodies to identify their cytokine secretion profiles. How closely the garnered information from these studies will relate to *in vivo* cellular function remains unclear. Although each technique will yield important discoveries, none can perfectly decipher the *in vivo* pathogenic immune response.

With these limitations in mind, we have focused on developing new methods to characterize immune responses from whole tissue RNA-Seq (12, 62). We view this strategy to be an important complement to work currently being conducted by other investigators. The main advantage being that the data is not subject to experimentally-induced changes in gene expression. Its main disadvantage, however, is that it cannot discriminate between direct or indirect correlations between genes of interest.

In our current study, we utilize a machine-learning 2D visualization strategy, t-SNE, to characterize *IL17A, IL22*, and *IL23A* gene expression in the context of the entire psoriatic transcriptome. The 2D map of the psoriatic transcriptome revealed distinct gene clusters corresponding to common immune cell types (e.g., B cells, T cells, neutrophils).

Our data did not support the existence of a dual-secreting IL-17A/IL-22 Th17 cell as the major source of these cytokines in psoriasis. In fact, in the 2D model, these genes are located far from one another. As such, *IL22* correlated with several genes that did not appear to have a relationship with *IL17A*. In addition, a set of genes identified to be involved in psoriasis pathophysiology (*CARD14, CXCL5, CXCR2, DDX58, IFIH, PTPN22*, and *TNFRSF9*) correlated with *IL17A* and *IL23A*, but did not correlate with *IL22*.

Though, IL-22 is commonly considered a hallmark Th17 cytokine (63), our results are in line with studies demonstrating the existence of uniquely secreting IL-17 and IL-22 T cells or the existence of other cytokine-secreting phenotypes (48, 64–69), although these other studies usually relied upon non-physiologic *ex vivo* T cell stimulation. Another possibility is that other cell types, such as γδ T cells or mast cells, contribute to the IL-22 production in psoriasis (48, 65, 70). Even neutrophils have been implicated as major producers of IL-22 and IL-17A (71) and recent animal models have re-explored their role as effector cells in psoriasis pathophysiology (22, 72, 73). Indeed, there are numerous studies supporting a key function of these cells (71, 74–78). Single cell sequencing may provide information to verify the relationship between *IL17A* and *IL22* expression. Although it is possible that dual-secreting IL-17A/IL-22 Th17 cells exist, our results suggest that they are not a major source of IL-22.

Although we did not find evidence for a strong link between *IL22* with *IL17A* or *IL23A*, our results do support a strong correlation between the expression of *IL22* and the keratin genes, such as *KRT6A, KRT6B, KRT6C, KRT16, KRT17*, a finding in accord with IL-22's ability to induce epidermal hyperproliferation (48). IL-22 is clearly a major cytokine involved in psoriasis pathophysiology. In animal models, it has been demonstrated to simulate psoriasis-like epidermal changes (47, 79) and elevated levels of IL-22 positively correlate with disease severity in humans, as measured by Psoriasis Area Severity Index (PASI) scores (80–83).

## Conclusion

Although dual-secreting T cells may exist, our results demonstrate that it is unlikely that the classical Th17 cells (IL-17A/ IL-22 dual-secreting T cells) play a universal role in psoriasis pathophysiology. RNA-Seq analysis revealed that the expression of these cytokines seems to be largely unrelated to one another in the psoriasis transcriptome. However, the expression of *IL17A* did correlate with *IL23A* but, interestingly, unique relationships between *IL23A* and genes unrelated to *IL17A* were also established, supporting a broad function of IL-23.

Taken together, these results do not support the current dogma that IL-17A/IL-22 dual-secreting Th17 T cells are the major driver of psoriasis pathophysiology. In addition, our results support unique functions of IL-23 that are unrelated to its known role in supporting Th17 responses. Finally, we demonstrate that the expression of genes linked to psoriasis susceptibility also correlate with expression of either *IL17A, IL22*, or *IL23*. This supports the aforementioned cytokines' involvement in multiple avenues of psoriasis susceptibility.

2D mapping of inflammatory transcriptomes is an exciting innovative modality that may help us visualize relationships of all genes expressed in a disease process. When applied to gene expression relationships in psoriatic lesional skin, distinct clusters of cell lineage genes could be identified, supporting the presence of a complex crosstalk among separate cell lines in disease development. In the near future, single cell transcriptome analysis will provide additional insight into psoriasis pathogenesis. Identifying the cells responsible for the psoriasis phenotype will bring us one step closer to developing a cure for psoriasis.

## Data Availability

Publicly available datasets were analyzed in this study. This data can be found here: “http://www.ncbi.nlm.nih.gov/Traces/sra”.

## Author Contributions

EM, AAM, AIM, GL, MS, SR, and SH contributed to the conception and design of the study; LT and JG organized the database; AAM performed the statistical analysis; SL, JW, and CA performed data mining, SL and EM wrote the first draft of the manuscript; SL, IA, and EM wrote sections of the manuscript. All authors contributed to manuscript revision, read and approved the submitted version.

### Conflict of Interest Statement

The authors declare that the research was conducted in the absence of any commercial or financial relationships that could be construed as a potential conflict of interest.

## Supplementary Material

Supplemental Figure 1 Funnel plot representation demonstrating RNA-seq datasets analyzed in the meta-analysis of *IL17A* and *IL22*. All data points, representing individual data sets, fall within the 95% confidence interval. In our meta-analysis, the *p* value for residual heterogeneity did not reach significance (*p* = 0.34), indicating that all datasets are within the variation that is expected for this particular meta-analysis.

Supplemental Figure 2 Additional genes that positively correlate with *IL22* expression.
