# Supplementary figures and images for: 2D Visualization of the Psoriasis Transcriptome Fails to Support the Existence of Dual-Secreting IL-17A/IL-22 Th17 T Cells
Source: Front Immunol. 2019 Apr 4;10:589. doi: 10.3389/fimmu.2019.00589 (PMC6458264; doi:10.3389/fimmu.2019.00589)

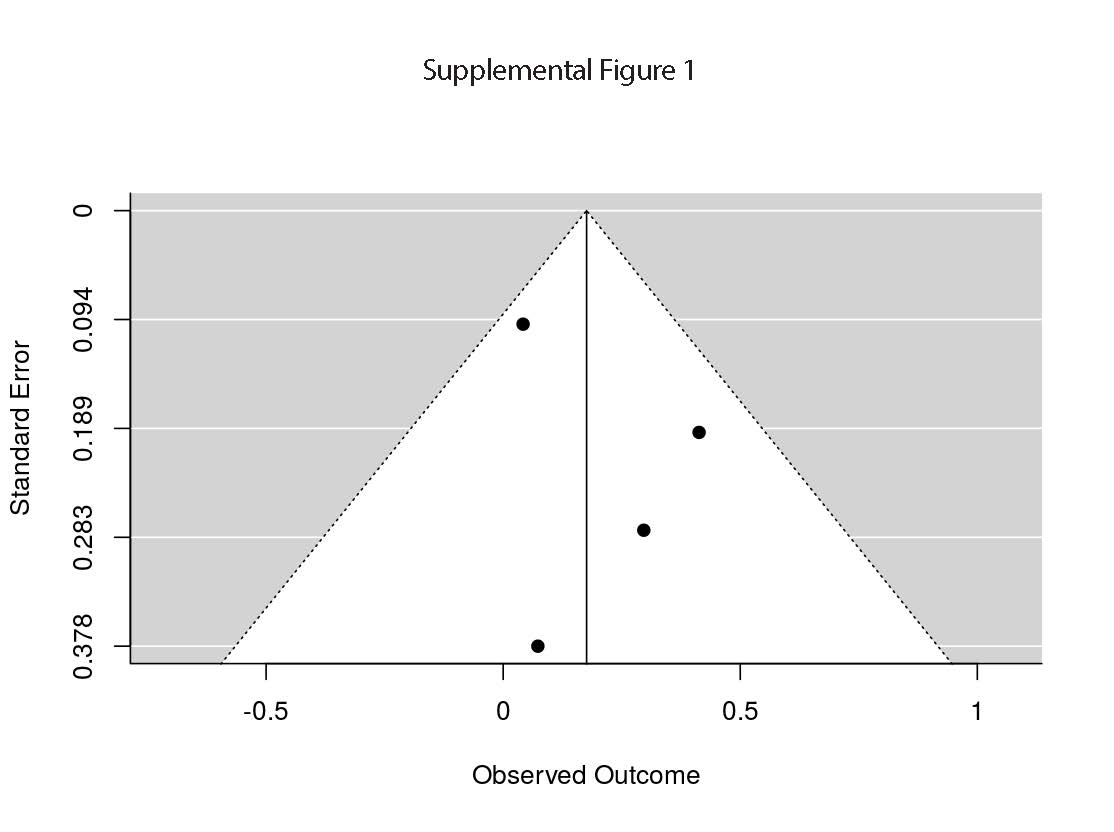

Supplement: Supplemental Figure 1 — Funnel plot representation demonstrating RNA-seq datasets analyzed in the meta-analysis of IL17A and IL22. All data points, representing individual data sets, fall within the 95% confidence interval. In our meta-analysis, the p value for residual heterogeneity did not reach significance (p = 0.34), indicating that all datasets are within the variation that is expected for this particular meta-analysis. [file Image_1.jpg]

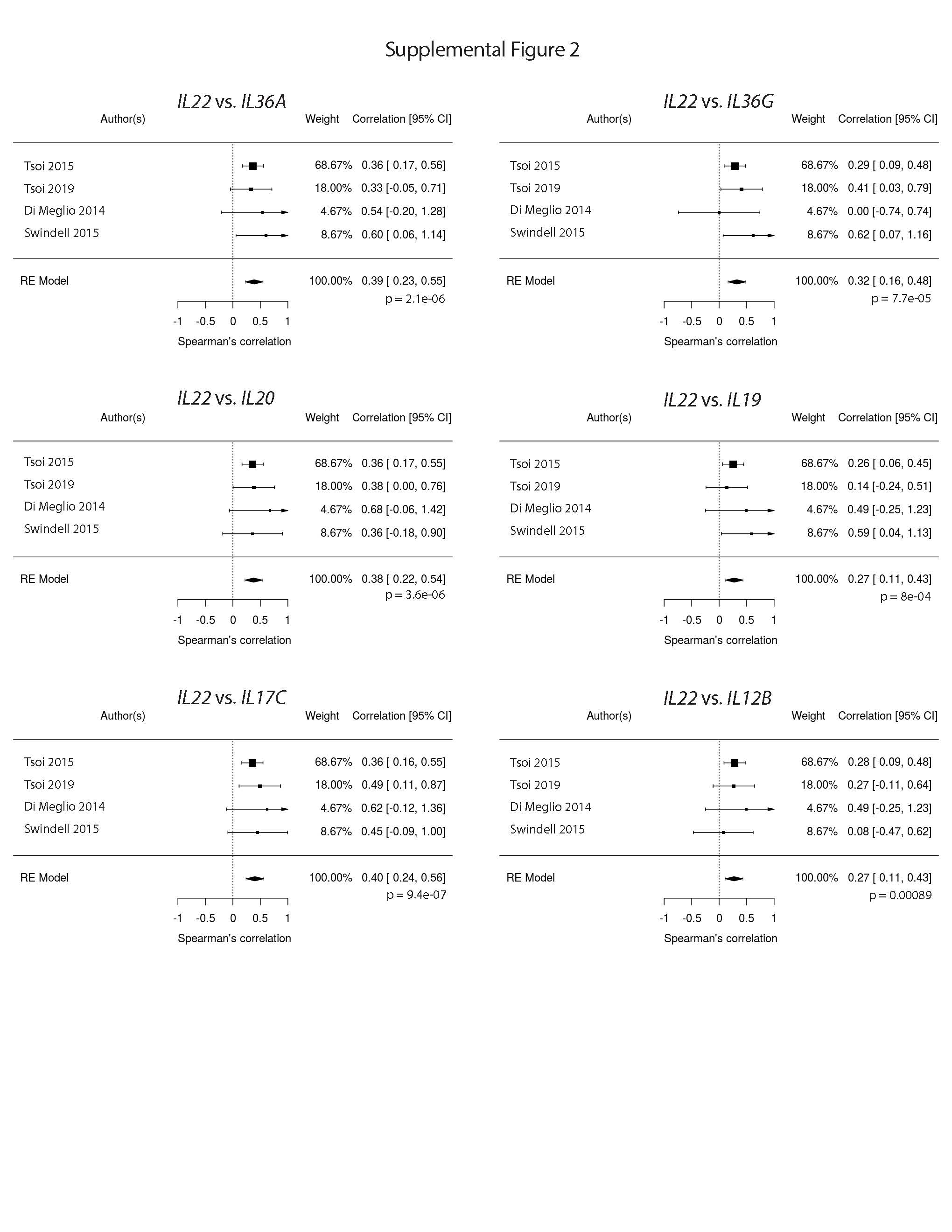

Supplement: Supplemental Figure 2 — Additional genes that positively correlate with IL22 expression. [file Image_2.jpg]
